# Boerhaave’s syndrome: Thoracolaparoscopic approach

**DOI:** 10.4103/0972-9941.68585

**Published:** 2010

**Authors:** Shulmit Vaidya, Suraj Prabhudessai, Nitish Jhawar, Roy V Patankar

**Affiliations:** Department of Gastrointestinal and Minimally Access Surgery, Joy Hospital, Mumbai, India

**Keywords:** Boerhaave’s, thoracoscopy, feeding jejunostomy, laparoscopy, oesophagostomy

## Abstract

We present a case of Boerhaave’s syndrome managed thoracolaparoscopically. A 45-year- old man presented with hydropneumothorax following severe retching. He was treated with Intercostal drainage insertion as the primary management and referred to a tertiary care centre. There endoscopic stapling was attempted, following which he developed a leak. He presented to us with severe sepsis and mediastinal collection on the ninth day following the perforation. We treated him with thoracoscopic mediastinal toilet, laparoscopic-assisted feeding jejunostomy and cervical oesophagostomy. The patient was managed conservatively. A computed tomography (CT) scan was repeated at intervals of 15 days. He was continued on full jejunostomy feeds. Regular assessment of the oesophagus injury was conducted via the CT scan. The patient had complete healing of the perforation at end of two months. His oesophagostomy was closed and he remained symptom-free at follow-up. We conclude that thoracoscopy has an important role to play in the management of patients with mediastinal sepsis and late presentation of Boerhaave’s perforation.

## INTRODUCTION

Barogenic rupture of the oesophagus is uncommon. Mediastinal sepsis is the most common cause of mortality. One hundred per cent mortality exists without intervention and overall survival with surgery is only 70%. Immediate surgery is the treatment of choice. In patients presenting late, external drainage of the thorax, care of nutrition and proximal diversion are used. We present a novel approach consisting of thoracoscopic mediastinal toilet, laparoscopic-assisted feeding jejunostomy and cervical oesophagostomy in the patient reported here.

## CASE REPORT

**Figure 1:**
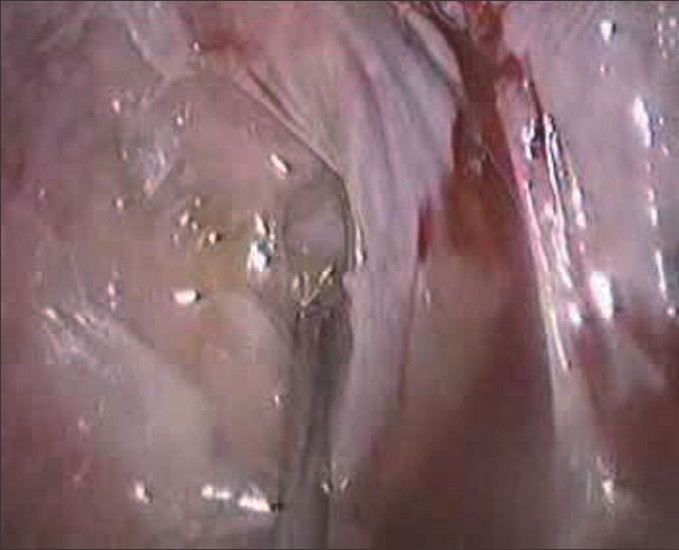
Barium swallow

**Figure 2:**
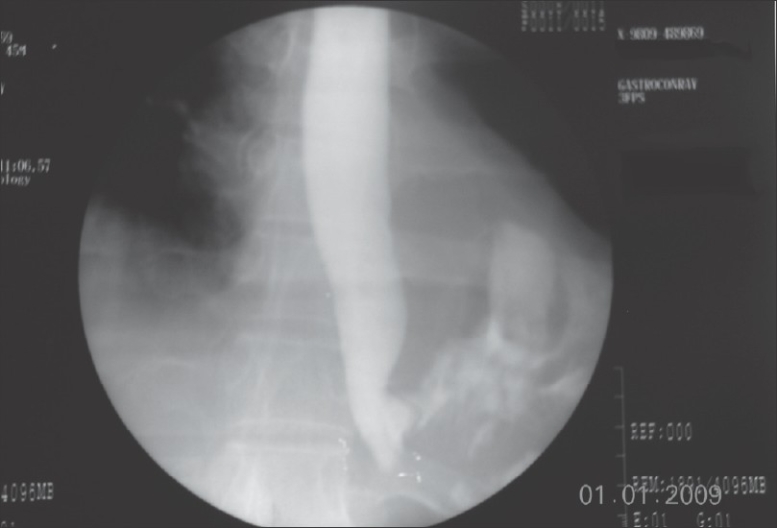
Thoracoscopic view of the cavity

**Figure 3:**
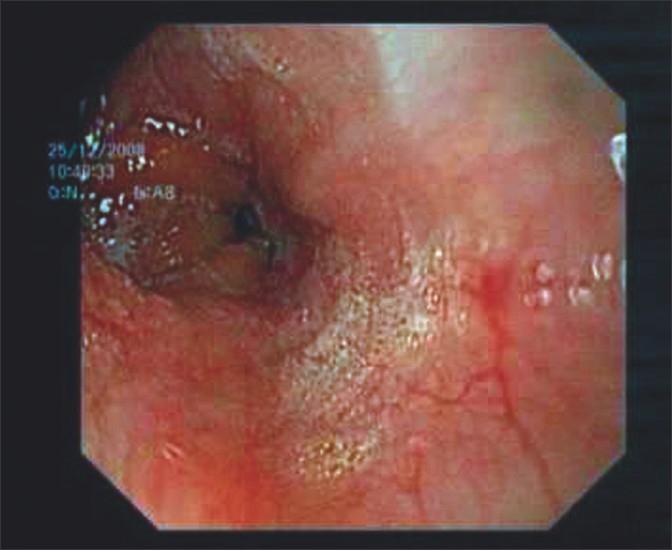
Healed perforation on OGD scopy

A 45-year-old male presented to our hospital, having been previously treated at another institute. Spme nine days prior, he had developed severe chest pain following retching. A chest x-ray then had showed a hydropneumothorax. Emergency intercostal drainage (ICD) insertion was done and a water-soluble contrast study had showed a leak from the lower oesophagus [Figure 1]. An endoscopic stapling was performed at the other institution where he was managed and that leaked on the third postoperative day. The patient presented to us with septic shock on day nine following the original perforation. A CT scan showed a free leak into the pleural space with a large collection. A new ICD was inserted, but it failed to clear the pleural space. We then decided to proceed with thoracoscopic mediastinal toilet. The patient was administered general anaesthesia with a double-lumen endotracheal tube. He was placed in the lateral decubitus position. A thoracic epidural catheter was placed for postoperative analgesia. A working port was established in the sixth intercostal space in the midaxillary line and two other 5 mm ports were placed. A 30 degree telescope was used for the procedure. A thorough mediastinal toilet was given [Figure 2]. The site of the perforation was identified but was too friable for attempting any form of closure. Local decortication was done. The thorax was drained with two IC drains. A laparoscopic-assisted feeding jejunostomy was carried out. At the end of the procedure, a left-sided cervical loop oesophagostomy was performed. The entire procedure lasted 150 minutes and blood loss was approximately 300 cc. No ventilatory support was required postoperatively. Jejunostomy feeds were started on the second postoperative day. On the fifth postoperative day, the patient was off IV antibiotics and on full jejunostomy feeds. During the course in the hospital he developed lower zone consolidation, which was treated with aggressive chest physiotherapy and IV antibiotics. The cervical oesophagostomy was closed two months after endoscopic [Figure 3] and radiological confirmation of healing of perforation.

## DISCUSSION

Boerhaave first described spontaneous rupture of the oesophagus in 1724. It typically occurs after forceful emesis. Boerhaave syndrome is a transmural perforation of the oesophagus, to be distinguished from Mallory-Weiss syndrome, a nontransmural oesophageal tear, also associated with vomiting. As it is usually associated with emesis, Boerhaave syndrome is not truly spontaneous. However, the term is useful for distinguishing it from an iatrogenic perforation, which accounts for 85 – 90% of the cases of oesophageal rupture.

Diagnosis of Boerhaave syndrome can be difficult because often no classic symptoms are present and delays in presentation for medical care are common. Approximately one-third of all cases of Boerhaave syndrome are clinically atypical. Prompt recognition of this potentially lethal condition is vital to ensure appropriate treatment. Mediastinitis, sepsis and shock are frequently seen late in the course of the illness, which further confuses the diagnostic picture.

Reported mortality estimate is approximately 35%, making it the most lethal perforation of the gastrointestinal (GI) tract. The best outcomes are associated with early diagnosis and definitive surgical management within 12 hours of the rupture. If intervention is delayed for longer than 24 hours, the mortality rate (even with surgical intervention) rises to higher than 50% and to nearly 90% after 48 hours. Left untreated, the mortality rate is close to 100%.

Boerhaave’s syndrome is a barogenic injury to the oesophagus resulting from a sharp increase in intraluminal pressure during vomiting against a closed cricopharyngeus.[1] The most common site of the tear is the left posterolateral wall of the lower third of the oesophagus, with a leak into the left pleural cavity. However, rupture of the middle third of the oesophagus opens into the right pleural cavity.[2] It is commonly seen in middle-aged men, usually with excessive dietary or alcoholic intake. The classical presentation is with vomiting and lower chest pain. The presence of subcutaneous emphysema with the two above-mentioned symptoms constitute Mackler’s triad.[3] Other findings include tachypnoea, dyspnoea, tachycardia, fever, hypotension and abdominal symptoms. Chest examination reveals findings of effusion or pneumothorax and, as seen in our patient, an intercostal tube insertion is often carried out prior to the diagnosis. Hamman crunch (mediastinal crackling sound) may be heard due to pneumomediastinum. Septic complications ensue with progress of disease. A chest radiograph confirms the presence of hydropneumothorax, needing intercostal drainage. Other findings in the chest x-ray are pneumomediastinum, meditational widening, subcutaneous emphysema and V-sign of Naclerio (retrocardiac streaks of air forming V). The characters of pleural fluid include a pH of < 6, undigested food particles and a high amylase content.[4] Contrast studies and CT scan have a limited role. Endoscopy is both diagnostic and therapeutic as mentioned below. Management depends upon the time of diagnosis from the original incident and overall medical condition of the patient. Nonsurgical treatment has practically no role in managing patients with oesophageal perforation. It is advised only in those with late presentation or in those with poor medical reserve.[5] We opted for conservative treatment due to the lack of consent for an early surgical intervention. This includes keeping the patient on parenteral hyperalimentation with total prohibition of oral feeds, broad-spectrum antibiotics and adequate drainage of pleural cavity, with tube thoracostomy. Surgery forms the mainstay of treatment and is indicated in all patients. Oedematous, stiff and friable wound edges make primary repair after 24 hours technically difficult, David *et al*. advocates primary repair even for perforations as old as 72 hours.[6] Repair of the perforation includes a primary closure along with reinforcement with viable tissue graft wraps such as, omental, intercostal muscle pedicle, diaphragmatic, pleural pedicled flaps, or pericardial fat pad.[7] This is necessary as necrosis, inflammation and oedema at the site of the rupture compromises the delicate blood supply of the oesophagus. Useful adjuncts are oesophagostomy and gastrostomy for drainage and a feeding jejunostomy. Therapeutic endoscopy offers promising results. Endoscopic placement of self-expanding metallic stents offers limited invasiveness and good results even in patients with extreme delay in diagnosis. Stents can also be placed after a failure of conservative treatment or when associated with unresectable malignancies. Placement of a self-expanding mesh stent is an attractive option and can be placed at the time of initial presentation itself.[8] Patients with a delayed presentation are managed with a T-tube, to create a controlled oesophagocutaneous fistula, which is closed after initial stabilization.[9] Oesophagectomy as a last resort is useful in complicated oesophageal ruptures.

There have been a few reports of Boerhhave’s syndrome being managed successfully by thoracoscopic approach. Scott *et al* were the first to report a minimally invasive approach to this condition as early as 1995.[10] Landen *et al* treated three patients of Boerhhave’s syndrome laparoscopically.[11] One patient had a transhiatal laparoscopic closure of the perforation, one had a closure reinforced by a fundal patch and another had fundic patch alone. One of their patients had a second perforation of the proximal esophagus, which was sutured through a cervical incision. Mediastinal debridement was performed transhiatally and also by means of a mediastinoscope introduced via the cervical incision in one patient. One patient required secondary thoracoscopic debridement of a pleural empyema but died of sepsis after 1 month. Dapri and colleagues found the prone position useful for thoracoscopic debridement in a patient with delayed diagnosis of Boerhaave’s syndrome.[12]

In conclusion, Boerhaave’s syndrome is an uncommon life threatening condition demanding early diagnosis with a high index of suspicion. Urgent surgical treatment is indicated in all patients. Even if the initial surgery is delayed or has failed, as in our patient, an aggressive approach still helps reduce the morbidity and associated mortality in this condition. In experienced hands minimally invasive surgery forms a safe and valid option for treatment of this challenging problem.
